# Sirt2-associated transcriptome modifications in cisplatin-induced neuronal injury

**DOI:** 10.1186/s12864-020-6584-2

**Published:** 2020-03-02

**Authors:** Xin Zhao, Wuying Du, Manchao Zhang, Zainab O. Atiq, Fen Xia

**Affiliations:** 1 Department of Radiation Oncology, University of Arkansas for Medical Sciences, Little Rock, AR 72205 USA; 2 Institute for Systems Genetics, NYU Langone Health, New York, NY 10016 USA

**Keywords:** RNAseq, Cisplatin, Neuropathy, Bioinformatics, *Sirt2*

## Abstract

**Background:**

Chemotherapy-induced peripheral neuropathy is not only one of the most common causes of dose reduction or discontinuation of cancer treatment, but it can also permanently decrease the quality of life of cancer patients and survivors. Notably, *Sirt2* protects many organs from various injuries, including diabetic peripheral neuropathy. As demonstrated previously by our laboratory and others, the overexpression of *Sirt2* can improve cisplatin-induced neuropathy, although the mechanism is still unclear.

**Results:**

In this study, the underlying mechanism by which *Sirt2* protects neurons from cisplatin-induced injury was explored using the RNAseq technique in cultured rodent neurons. *Sirt2* status was modified by genetic knockout (*Sirt2*/KO) and was then reconstituted in *Sirt2*/KO cells (*Sirt2*/Res). We observed 323 upregulated genes and 277 downregulated genes in *Sirt2*-expressing cells (*Sirt2*/Res) compared to *Sirt2*-deficient cells (*Sirt2/*KO). Pathway analysis suggested that *Sirt2* may affect several pathways, such as MAPK, TNF, and cytokine–cytokine interaction. Furthermore, cisplatin-induced changes to the transcriptome are strongly associated with *Sirt2* status. Cisplatin induced distinctive transcriptome changes for 227 genes in *Sirt2*-expressing cells and for 783 genes in *Sirt2*-deficient cells, while changes in only 138 of these genes were independent of *Sirt2* status. Interestingly, changes in the p53 pathway, ECM–receptor interactions, and cytokine–cytokine receptor interactions were induced by cisplatin only in *Sirt2*-deficient cells.

**Conclusions:**

This study demonstrated that *Sirt2* regulates the transcriptome in cultured rodent neuronal cells. Furthermore, *Sirt2*-associated transcriptome regulation may be an important mechanism underlying the role of *Sirt2* in organ protection, such as in cisplatin-induced neuronal injury. *Sirt2* may be a potential target for the prevention and treatment of chemotherapy-induced neuropathy.

## Background

Cisplatin is a common and effective chemotherapeutic agent used to treat many types of cancers. Peripheral neuropathy during cisplatin treatment is a common, significant, and dose-limiting side effect [1]. This cisplatin-induced neuropathy not only significantly impacts the patient’s quality of life, but also impedes cancer control. According to a survey of patients treated with cisplatin, non-symptomatic and symptomatic neuropathy could be found in 38 and 28% of patients with non-seminomatous testicular cancer, respectively, and 6% were found to have disabling polyneuropathy [2]. In rodent models, cisplatin-induced neuropathy presents with mechanical allodynia, spontaneous pain, and numbness [3, 4].

One of the mechanistic models for cisplatin-induced peripheral neuropathy is dorsal root ganglion (DRG) sensory nerve injury [5, 6]. It has been suggested that mitochondrial dysfunction, in particular the abnormality of axonal mitochondria [7, 8]; changes in sensory neuron signaling cascades; calcium homeostasis; and oxidative stress may contribute to cisplatin-induced neuropathy [1]. However, the underlying cause and pathological mechanism of cisplatin-induced neuropathy is still unclear.

As a member of the sirtuin family of proteins, *Sirt2* functions as an NAD^+^-dependent deacetylase. *Sirt2* expression is higher in olfactory sensory neurons than in neighboring cells; when compared with *Sirt1*, *Sirt2* expression was higher in injured retinal ganglion cells in the optic nerve [9, 10]. Additionally, *Sirt2* was recently found to be a required target for neurite outgrowth. Knockdown of *Sirt2* resulted in a deficiency in the LKB-1/AMPK/PGC-1α pathway and subsequent mitochondrial dysfunction and peripheral neuropathy in type 1 diabetic rodents [11]. Low expression of *Sirt2* was also found in the diabetic cortex, which induced oxidative stress and diabetic neuropathy [12]. Furthermore, expression quantitative trait loci were used to demonstrate that *Sirt2* is associated with thermal nociception [13]. Consistent with previous reports that *Sirt2* overexpression could alleviate neuropathic pain in the chronic constriction injury model via deacetylation of nuclear factor-kappa B (NF-κB) [14], our recent work also demonstrates that *Sirt2* protects against cisplatin-induced neuropathy in mice (manuscript submitted).

While there is a substantial amount of literature indicating a protective role for *Sirt2* in cisplatin-induced neuropathy, the mechanism by which *Sirt2* protects against cisplatin-induced neuropathy is unknown. Whether or not *Sirt2* is involved in regulating the transcriptome has yet to be determined. In this study, we explored the underlying mechanism of *Sirt2* in protecting neurons from cisplatin-induced injury using RNAseq in cultured rodent neurons.

## Results

### Summary of RNAseq

**Fig. 1:**
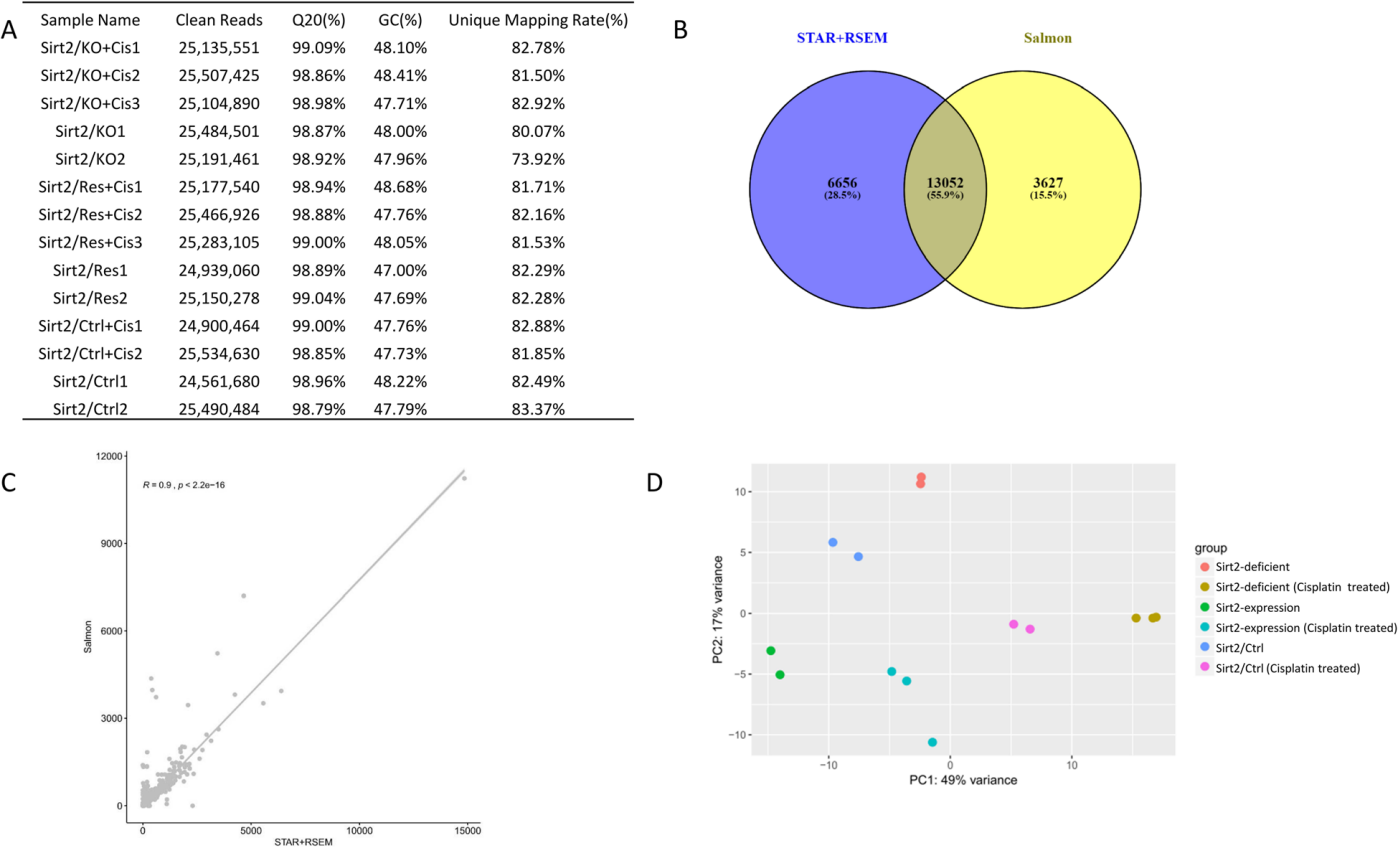
RNAseq data. **a** Details of RNAseq parameters with clean-read numbers, Q20%, GC content, and unique mapping rate. **b** Venn diagram showing number of genes detected using two different quantification methods. Numbers on the left were obtained with STAR+RSEM; numbers on the right were obtained with Salmon. **c** Pearson correlation between the two different quantification methods. **d** PCA analysis of a total of 14 samples after dropping the 4 technical outliers

To explore the underlying molecular mechanisms involved in *Sirt2* function in cisplatin-induced peripheral neuropathy, we studied the *Sirt2*-associated and cisplatin-induced differential gene expression profiles using RNAseq data analysis in cultured neuronal cells derived from rat DRG sensory neurons. Eighteen samples were divided into 6 groups according to genotype and treatment, and each experiment was performed in triplicate (Genotype: Sirt2 genetic knockout (Sirt2/KO) and was then reconstituted in Sirt2/KO cells (Sirt2/Res), Sirt2 normal expressed *(Sirt2*/Ctrl); Treatment: cisplatin or Phosphate-buffered saline. After removing the barcode, index, and adapters, approximately 25.21 million reads could be detected in each sample (Fig. 1a). The remaining 14 samples were used for further analysis. Alignment results from STAR+RSEM showed that the unique mapping rate was 81.55% on average, and 19,708 genes could be found when compared against the rat reference genome RGSC 5.0/rn5. We also used Salmon for the non-alignment quantification, and 28,421 transcripts and 16,679 genes were identified in this test. We found 13,052 overlapping genes between these two quantification tools (Fig. 1b). The technical repeats of *Sirt2*/Ctrl+Cis and *Sirt2*/Ctrl were analyzed to see which method was most effective, and the results showed that all three repeats had a Pearson’s correlation coefficient of *r* > 0.99 in both methods. A correlation of *r* = 0.9 and *P* < 1 × 10^− 16^ was also found between these two different methods (Fig. 1c). Based on this, we used the overlapping genes for the analysis that followed. The results of principal component analysis showed that no extra sub-cluster of other group was found (Fig. 1d).

### Differential gene expression correlates with *Sirt2* status

**Fig. 2:**
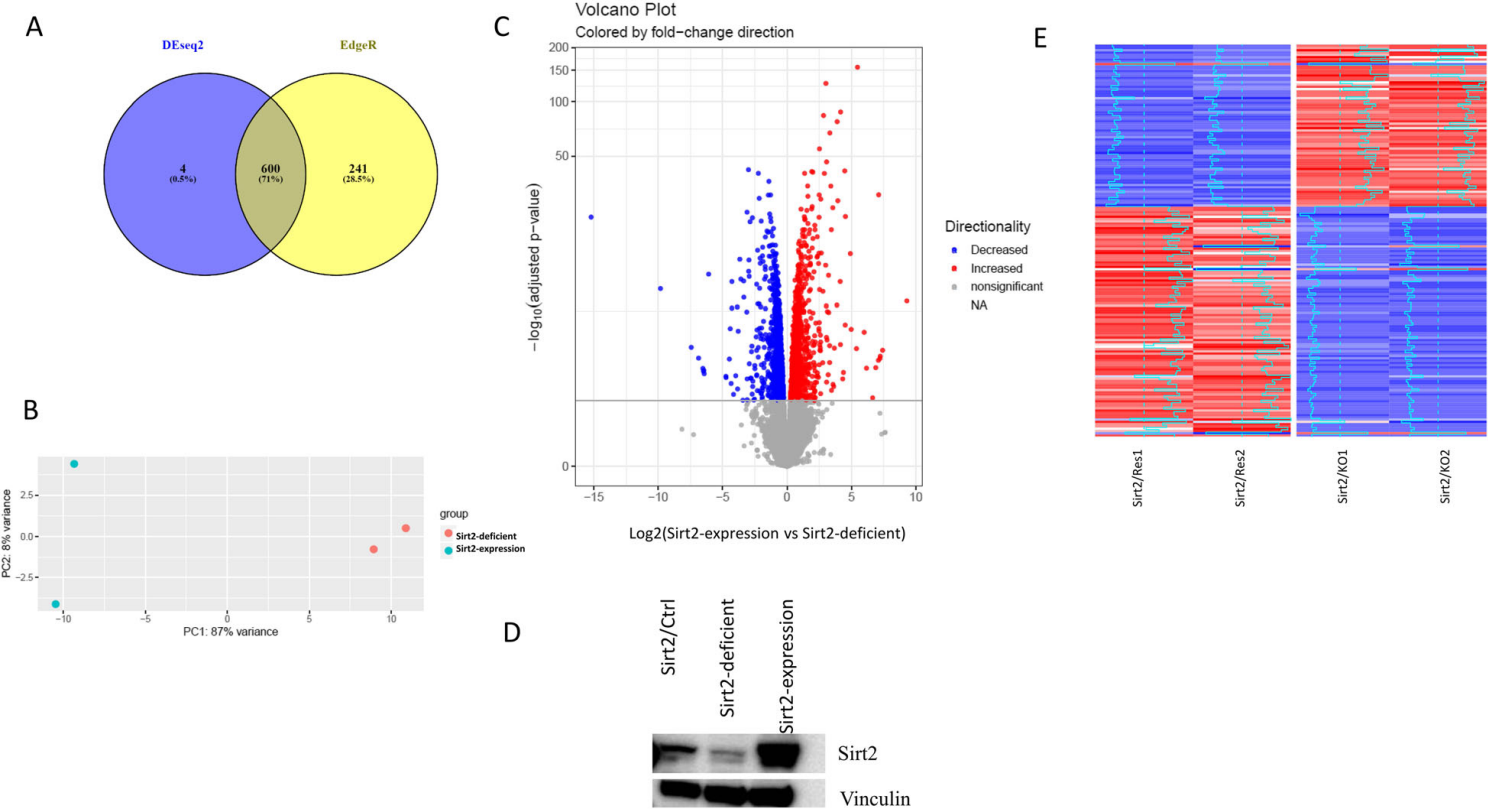
Differential transcriptomes in *Sirt2*-expressing and *Sirt2*-deficient cells. **a** Venn diagram showing calculated differentially expressed genes by Deseq2 and EdgeR. **b** PCA analysis for *Sirt2*-expressing and *Sirt2*-deficient samples. **c** Volcano plot of differentially expressed genes. Red represents an increase in expression; blue represents a decrease; and grey indicates no significant difference. **d** Western blot for Sirt2 in *Sirt2*/Ctrl, *Sirt2*-expressing, and *Sirt2*-deficient cells. Vinculin used as a loading control. **e** Heatmap of differentially expressed genes with fold change > 2, Red represents an increase in expression; blue represents a decrease

To increase the reliability of the analyzed results, two differential expression algorithms, edgeR and DEseq2, were used. With the cutoff false-discovery rate (FDR) < 0.1 and fold change > 2, 604 genes showed significant change of mRNA expression using DEseq2, and 841 genes showed significance using EdgeR; 600 genes showed significance in both methods and would be used for further analysis (Fig. 2a). Among these 600 significantly genes with expression changes, 323 genes were upregulated and 277 were downregulated in *Sirt2*-expressing cells compared to *Sirt2*-deficient cells (Fig. 2b, Fig. 2c). With the cutoff of FDR < 0.1 and fold change > 10, we found 30 genes upregulated and 25 genes downregulated in *Sirt2*-expressing cells when compared to *Sirt2*-deficient cells (Additional files 2).

To confirm *Sirt2* status in cultured rodent neuron cells, we used qPCR and western blot. Levels of *Sirt2* mRNA expression could still be detected in knockout cells, despite being 12.55-fold lower than levels in *Sirt2*-expressing cells (Fig. 2d). This may be due to a residual cell population in which *Sirt2* knockout was unsuccessful. Next, we used qPCR to verify the results of the algorithm. Three of the genes with the highest fold change and 4 of the genes with the lowest fold change were tested in 2 differentiated neuronal cell lines: 50B11 and PC12. With the fold change larger than 2, we verified 6 out of 7 genes in 50B11 and 5 out of 7 genes in PC12 (Table 1).

**Table 1:** Fold change by qPCR among different cell lines

Symbol	Res/KO RNAseq (FC)	Res/KO 50b11 (FC)	Res/KO PC12 (FC)
*Bmp7*	67.75	2.10*	3.21*
*Mycn*	63.48	2.11*	1.08
*Igfbp5*	44.27	0.85	1.37
*Abcc9*	0.01	0.23*	0.24*
*Kcnj8*	0.01	0.46*	0.1*
*Tceal3*	0.01	0.40*	3.96*
*Agtr2*	0.001	0.11*	2.39*

*FC* fold change. All genes were normalized to *Gapdh*. FC > 2 or < 0.5 considered significant. *P < 0.05

### Differential signaling pathways associated with *Sirt2* status

**Fig. 3:**
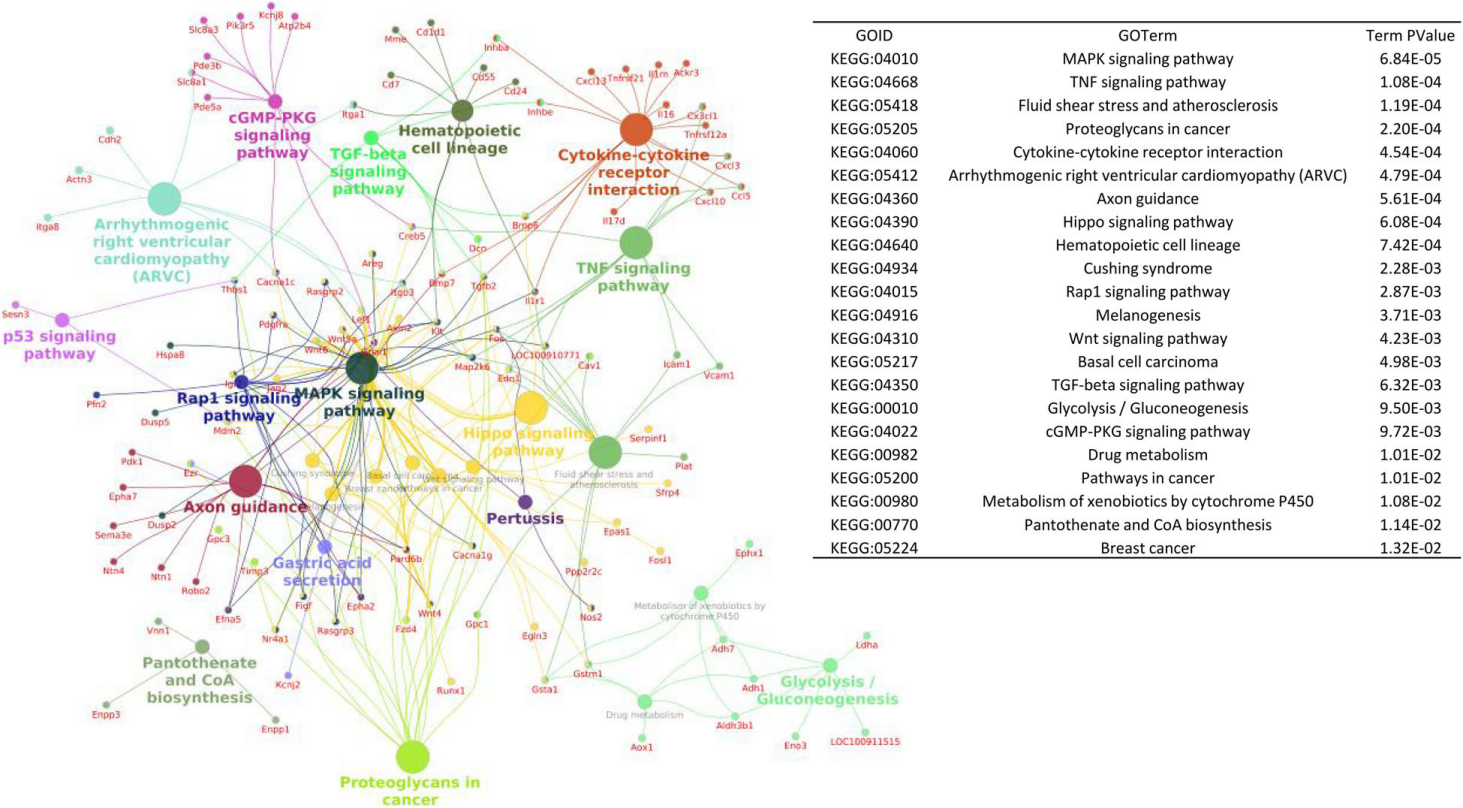
KEGG-based analysis for pathways potentially associated with Sirt2 status. Different colors in the network indicate different pathways. Names marked with red represent differentially expressed genes between *Sirt2*-proficient and *Sirt2*-deficient genotypes

Using ClueGo from Cytoscape, we next analyzed the 600 genes whose change of expression identified as significantly associated with *Sirt2* status (Fig. 2a). We observed more than 20 pathways that were significantly affected between *Sirt2*-expressing and *Sirt2*-deficient cells (Fig. 3); these pathways include MAPK (*P* = 6.84 × 10^− 05^), TNF (*P* = 1.08 × 10^− 04^), fluid shear stress and atherosclerosis (*P* = 1.19 × 10^− 04^), proteoglycans in cancer (*P* = 2.20 × 10^− 04^), and cytokine–cytokine receptor interaction (*P* = 4.54 × 10^− 04^).

### Sirt2 influences the transcriptome following cisplatin treatment

We analyzed the effects of *Sirt2* on gene expression in response to cisplatin in *Sirt2*-expressing and *Sirt2*-deficient 50B11 cells. With the cutoff of fold change >2 and FDR < 0.1, we found that cisplatin treatment in *Sirt2*-expressing cells upregulated 29 genes and downregulated 58 genes. In contrast, cisplatin treatment in *Sirt2*-deficient cells upregulated 528 genes and downregulated 115 genes. There were 138 genes induced by cisplatin independent of *Sirt2* status. To verify the analyzed results of the algorithm above, we used qPCR to examine the expression of 9 genes in *Sirt2*-expressing cells (3 upregulated genes and 6 downregulated genes); 7 out of 9 cisplatin-induced genes were verified in *Sirt2*-expressing cells, and 8 out of 9 genes were verified in *Sirt2*-deficient cells (Table 2).

**Table 2:** Fold change by qPCR of cisplatin-induced transcriptome in association with *Sirt2*

Symbol	*Sirt2-*expressing (treated vs untreated)		*Sirt2*-deficient (treated vs untreated)	
	RNAseq (FC)	qPCR (FC)	RNAseq (FC)	qPCR (FC)
*Klhdc8a*	10.09	2.59*	5.33	2.47*
*Bmp7*	5.22	2.57*	3.15	2.30*
*p21*	3.43	5.23*	5.15	5.45*
*Mycn*	0.88	0.72	0.32	0.47*
*Igfbp5*	0.47	0.38*	0.68	0.45*
*Abcc9*	0.44	0.38*	0.45	0.29*
*Slc2a13*	0.32	0.48*	0.25	0.37*
*Tceal3*	0.22	0.63	2.02	1.94*
*Kcnj8*	0.153	0.36*	7.63	1.69

*FC* Fold change. Untreated *Sirt2-*expressing and *Sirt2-*deficient were used as a reference, respectively. All genes were normalized to *Gapdh*. FC > 2 or < 0.5 considered significant. *P < 0.05

### Cisplatin-induced differential signaling pathways which are associated with *Sirt2* status

**Fig. 4:**
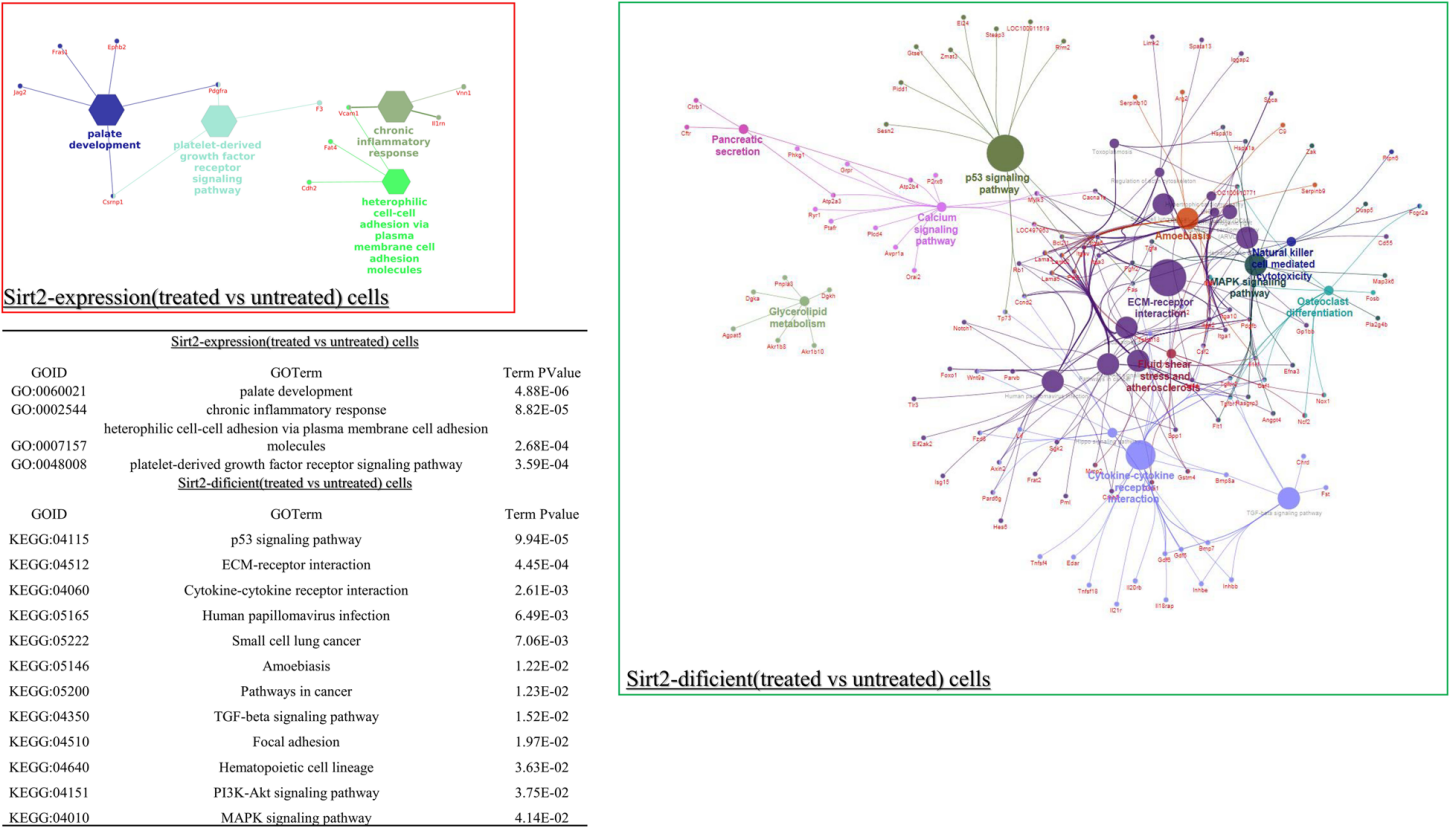
KEGG/David-based pathway analysis between *Sirt2-*proficient (treated versus untreated) (left) and *Sirt2*-deficient cells (treated versus untreated) (right). Different colors in the network indicate different pathways. Names marked with red represent differentially expressed genes found in each comparison

**Fig. 5:**
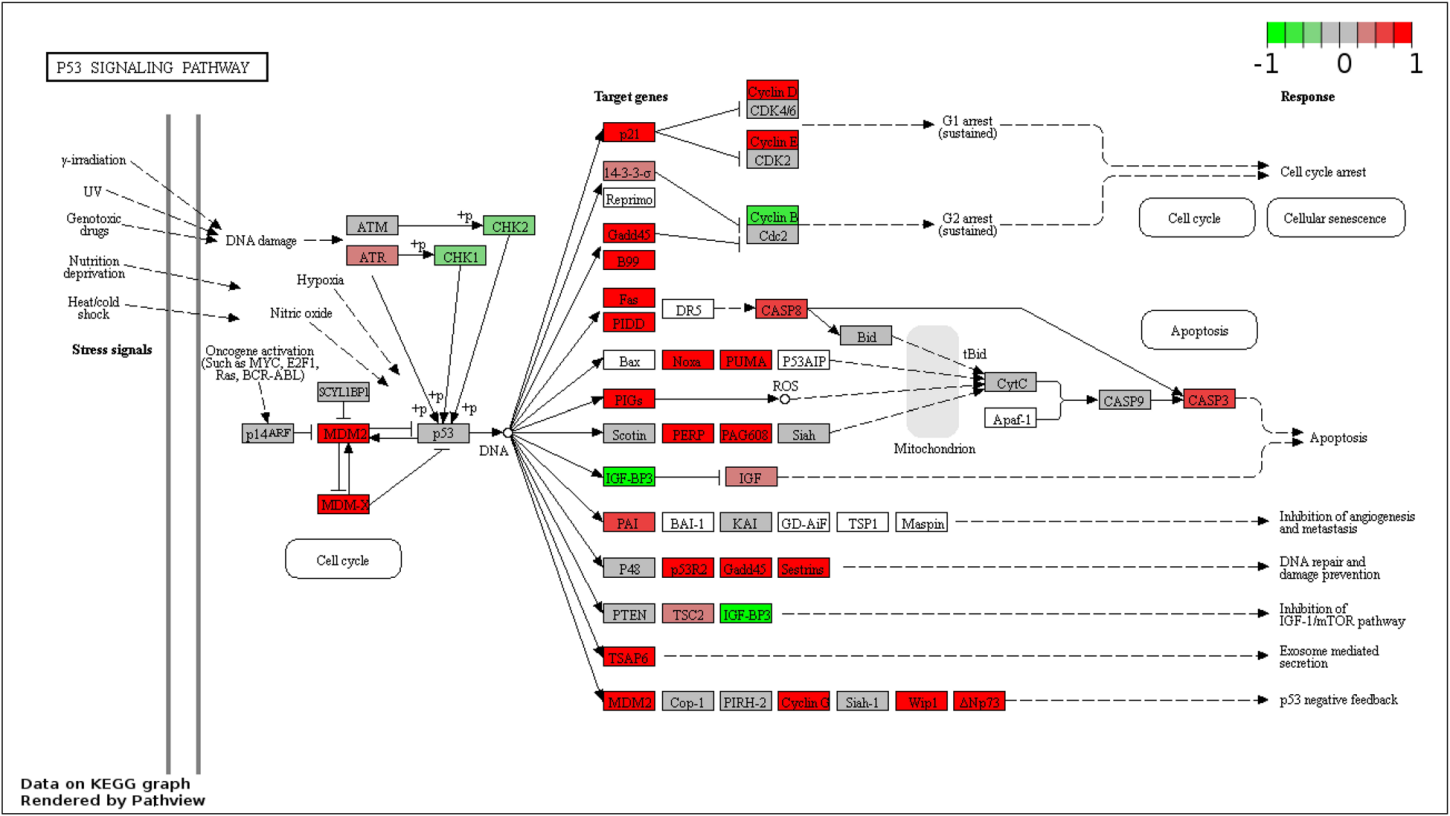
Cisplatin-induced P53 signaling pathway genes are differentially expressed between *Sirt2*-deficient and *Sirt2*-proficient cells. Red indicates higher expression and green indicates lower expression when comparing between *Sirt2*-deficient and *Sirt2*-proficient cells

Pathway analysis showed a significant difference of cisplatin-induced pathways dependent on *Sirt2* status (Fig. 4). Fewer pathways were induced by cisplatin in *Sirt2*-expressing cells than in *Sirt2*-deficient cells. Furthermore, the following pathways were induced only in *Sirt2*-deficient cells: p53, ECM–receptor interaction, and cytokine–cytokine receptor interaction pathways. Consistent with this, we also observed increased expression of downstream targets of p53 (Fig. 5), including *GADD45*, *GTSE1(B99)*, *FAS*, *PIDD*, *TP53I3*, *SERPINE1(PAI)*, *TSAP6*, and *MDM2*.

## Discussion

Cisplatin-induced neuropathy poses a major clinical challenge in cancer treatment. Much evidence suggests that *Sirt2* protects tissues from injury, including neuropathy resulting from various insults. The underlying mechanism by which *Sirt2* protects neurons from cisplatin-induced neuropathy is not clearly understood. In this study, we used RNAseq to examine differentially expressed genes in rodent neuronal cells with various *Sirt2* genotypes in the presence and absence of cisplatin. There was a noteworthy effect of *Sirt2* on the transcriptome and various signaling pathways. We observed 323 upregulated genes and 277 downregulated genes in *Sirt2*-expressing cells (*Sirt2*/Res) compared to *Sirt2*-deficient cells (*Sirt2*/KO). Pathway analysis suggests that *Sirt2* may affect several pathways, including MAPK, TNF, and cytokine–cytokine interaction. Furthermore, cisplatin-induced transcriptome changes are strongly associated with *Sirt2* status. Cisplatin induced distinct changes to the transcriptome for 227 genes in *Sirt2*-expressing cells and to 783 genes in *Sirt2*-deficient cells, while changes in only 138 of these genes were independent of *Sirt2* status. Interestingly, the following pathways were induced by cisplatin only in *Sirt2*-deficient cells: p53, ECM–receptor interaction, and cytokine–cytokine receptor interaction.

Other genes that have been verified by qPCR in current study may also affect neuronal function. Studies show that overexpression of bone morphogenetic protein 7 (*BMP7*) is beneficial in both nerves and Schwann cells after sciatic nerve injury, and it improves neuropathy in rats [15]. The *ABCC9* gene and its polypeptide product SUR2 are increasingly implicated in the aged human brain and induce the hippocampal sclerosis of aging [16]. As the receptor subtype of angiotensin II, *AGTR2* is expressed in sensory neurons and could be a potential target for nociception and neuronal regeneration [17]. *MYCN*, a member of *MYC* proto-oncogenes, expression levels may have a positive relationship with disease-free survival in astrocytoma and meningioma [18]. Also, potassium voltage-gated channel subfamily J member 8 (*KCNJ8*) may help to regulate blood pressure with the help of *ABCC9* [19].

*Sirt2* plays roles in many biological processes, including cell cycle regulation, tumorigenesis, neurodegeneration, lipid metabolism, and glucose metabolism [20]. One hypothesis is that *Sirt2* regulation of cisplatin-induced neuropathy is immune mediated. We showed that knocking out *Sirt2* induce changes in inflammatory pathways, including MAPK, cytokine-cytokine, and TNF pathways. Twenty-six genes were enriched in these three pathways; interestingly, all of these genes were upregulated in *Sirt2*-expressing cells by at least 2-fold (Supplemental Table 1). Additionally, some researchers suggested that *Sirt2* is involved in inflammatory pathways because *Sirt2*-KO mice had more morphological changes in microglia and an increase in pro-inflammatory cytokines in the brain [21]. Topical administration of a *Sirt2*-fused carrier protein to an inflamed ear reduced the levels of pro-inflammatory cytokines and inhibited the activation of NF-κB and MAPKs [22]. Additionally, another study showed that the MAPK pathway does function during platinum-induced neuropathy through the activation of p38 and ERK1/2 in DRG neurons [23].

The relationship between *Sirt2* and the calcium ion channel. The calcium signaling pathway could also explain *Sirt2*-related cisplatin sensitivity to some degree. Our previous proteomic data showed that *Sirt2*-overexpressing mice have increased protein levels of CAMK2A (FC = 1.55, *P* = 0.02), CAMK2D (FC = 1.39, *P* = 0.02), and CAMK2G (FC = 1.42, *P* = 0.03) when compared to *SIRT2-*deficient mice (manuscript submitted). We found that genes involved in calcium signaling (from KEGG) tend to be more highly expressed in *Sirt2*-expressing cells (Supplemental Table 2). We propose that this may be due to the impairment of the mitochondrial membrane caused by cisplatin. Mitochondrial dysfunction can lead to abnormal calcium uptake or leakage. To repair the harm caused by cisplatin, *Sirt2* might modify and strengthen the cisplatin-impaired calcium channel.

RNAseq is a credible method for studying global transcriptome regulation. However, a limited sample size can contribute bias to the results. Here, we performed qPCR to validate the RNAseq data. Because *Sirt2* has deacetylase activity, future research should use an increased sample size, focus on acetyl-proteomes, and evaluate acetylation levels combined with multi-omics, such as proteomics and transcriptomes.

## Conclusions

Using RNAseq to investigate the interaction of genotype with treatment, we demonstrated that the difference in *Sirt2* expression in cultured rodent neuronal cells leads to a significant change in the transcriptome with and without cisplatin treatment. These data suggest that transcription regulation could be an important mechanism underlying the protective function of *Sirt2* in cisplatin-induced neuropathy. In addition, *Sirt2* could potentially serve as a target in the treatment and prevention of cisplatin-induced neuropathy.

## Methods

### CRISPR-Cas9 gene editing

First, the single guide RNA (sgRNA) designed by the Atum bio CRISPR design tools, 5 pairs of sirt2 single guide RNA oligoes had been designed for the screening. The oligoes are designed based on the target site sequence (20 bp) and needs to be flanked on the 3′ end by a 3 bp NGG PAM sequence [24]. Lenti-CRISPR-v2 (AddGene 52,961) contains two expression cassettes, hSpCas9 and the chimeric guide RNA. The vector can be digested using BsmBI, and a pair of annealed oligoes can be cloned into the single guide RNA scaffold. Second, the cloned sgRNA Lenti-CRISPR-v2 vector sequencing by the hU6 promoter primer (5′-GAGGGCCTATTTCCCATGATT-3′) then make lentivirus. CMV-EGFP as a positive control for viral production. Lentivirus concentrated by 15,000 rpm spin 2 h in 4 °C [25]. 50B11 and PC12 cells infected by the concentrated lentivirus, 48 h later selected by puromycin 2μg/ml for another 48 h then harvest the cells detect the sirt2 expression by western blot. One of the five pairs was working perfect on rat SIRT2 gene (5′-GCGGAAGTCAGGGATTCCTG-3′).

### Cell culture, neuronal differentiation, and drug treatment

Cells were cultured at the University of Arkansas for Medical Sciences. 50B11 cells were from Dr. Youwei Zhang of Case Western Reserve University and PC12 (CRL-1721) was purchased from ATCC. Cultures were treated as follows: 50B11 cells were maintained in Dulbecco’s Modified Eagle’s medium (DMEM) supplemented with 10% FBS, 0.2 M glutamine, 1× B-27 supplement, 0.2% glucose, and 1% penicillin/streptomycin/glutamine and incubated at 37 °C with 5% CO_2_ [26]. The cells were cultured for at least 24 h, and confluence was allowed to reach 70% before differentiation. Forskolin (25 mM) was added directly to the culture medium for a final concentration of 75 μM. Full differentiation of neurite outgrowth appeared after 12 h.

PC12 cells were maintained in Dulbecco’s Modified Eagle’s medium (DMEM) supplemented with 5% calf serum, 5% horse serum and 1% penicillin/streptomycin/glutamine and incubated at 37 °C with 5% CO_2_.1% nerve growth factor was used for the cell differentiation. Cells were feed every other day with differentiating medium until ready for experiment.

Cisplatin was added to the culture medium at a final concentration of 2 μg/ml. After the 24 h, cells were harvested for RNA extraction. A flow chart of this process is shown in Supplemental Figure 1.

### RNA extraction

RNA was extracted according to TRIzol guidelines. All cells were lysed and homogenized by TRIzol and temporarily stored at − 80 °C; then, 0.3 ml chloroform was added per 1 ml of TRIzol reagent. The samples were mixed vigorously and centrifuged at 12,000×g for 15 min at 4 °C. RNA precipitated from the aqueous phase was mixed with 0.5 ml of isopropanol and centrifuged at 12,000×g for 30 min. The supernatant was removed, and 0.5 ml 75% ethanol was used for washing. The RNA concentration was determined with a NanoDrop One spectrophotometer.

### RNAseq and alignment

All RNA samples passed the threshold of quality control by the Agilent 2100 Bioanalyzer. The library preparation and RNAseq were processed at BGI-Hong Kong. The cDNA was synthesized and sheared into a 250-bp fragment, end-repaired, dA-tailed, and adaptor-ligated. After a 4-cycle PCR program, the libraries were sequenced on the BGI-SEQ500 platform with the 50-bp single-end sequencing strategy. Eighteen samples were divided into 6 groups with 3 repeats according to genotype and treatment, and 25 million reads were obtained for each sample (on average). All samples passed the quality control (QC) threshold of Q20 > 98%. After the first PCA and cluster analysis, 4 samples (1 *Sirt2*/Res, 1 *Sirt2*/KO, 1 *Sirt2*/Ctrl, and 1*Sirt2*/Ctrl+Cis) were eliminated as outliers. Fourteen samples that passed initial QC were used for further analysis (Fig. 1a). RGSC 5.0/rn5 RefSeq was downloaded from UCSC Genome Browser (http://genome.ucsc.edu). We used STAR 2.6.1b for the alignment [27]. FeatureCounts from the GATK4.0.8.1 package was used for the quantification [28]. RSEM 1.3.1 was used for the Transcripts Per Million (TPM) calculation with the parameters of “--star --estimate-rspd --append-names --output-genome-bam.” Salmon 0.11.3 was also used for the quantification of TPM with the parameters of “quant -i -l A --gcBias” [29]. The entirety of the pipeline can be seen in Supplemental Figure 2.

### Differentially expressed genes and pathway analysis

To analyze differentially expressed genes among the different genotypes and treatment conditions, we used DEseq2 and edgeR [30, 31]. During the procedure, the *P*-value (two-tailed) was calculated using a two-sample t-test. Corrections for false-positives (type I errors) were performed using Benjamin’s false discovery rate (FDR). We used “FDR < 0.1, Fold Change > 2 or < 0.5” as the threshold to judge the significance of differences in gene expression between the different conditions. All of the procedures were under the environment of R 3.4.3. Pathway analysis was performed using DAVID (http://david.abcc.ncifcrf.gov/), Gene Ontology (http://geneontology.org/), or the Kyoto Encyclopedia of Genes and Genomes (https://www.genome.jp/kegg/pathway.html). A hypergeometric test with the Benjamin and Hochberg false discovery rate (FDR) was performed using the default parameters to adjust the *P*-value. Cytoscape v3.6.1 and Pathview were used for pathway visualization [32, 33].

### Real-time quantitative PCR (qPCR)

The results of the RNAseq algorithm were verified by real-time quantitative PCR (qPCR, TaqMan). The top differentially expressed genes between *Sirt2-*proficient and *Sirt2-*deficient cells were chosen for verification. All TaqMan assays were purchased from ThermoFisher (*Abcc9*, Rn00464842_m1; *Bmp7*, Rn01528889_m1; *Mycn*, Rn01473353_m1; *Igfbp5*, Mm00516037_m1; *Tceal3*, Mm02747701_Sh; *Kcnj8*, Rn01492857_m1; *Agtr2*, Rn00560677_s1; and *Gapdh*, Rn01775763_g1). The TaqMan procedure was performed using an ABI StepOne system with 96-well plates as follows: 50 °C for 2 min to pre-heat the mixture, 95 °C for 20 s to activate the Taq polymerase, 40 cycles of 95 °C for 1 s, and 60 °C for 20 s. The qPCR data were analyzed with R 3.4.3. An unpaired t-test was performed to compare delta cycle threshold (dCt) values obtained in different groups. The data are expressed as relative fold-change based on the 2^- ΔΔCt^ method [34].

### Availability of data and materials

Raw sequence data were deposited at the European Nucleotide Archive (ENA) (http://www.ebi.ac.uk/ena/data/view/PRJEB36706).

## Supplementary information

**Additional file 1: Supplemental Figure 1.** Timeline of cell differentiation. Samples were divided into 6 groups (*Sirt2*/Res, *Sirt2*/Ctrl, *Sirt2*/KO, *Sirt2*/Res + Cis, *Sirt2*/Ctrl+Cis, or *Sirt2*/KO + Cis). Cells were differentiated with Forskolin for 12 h and treated with cisplatin for another 24 h. After that, RNA was harvested. **Supplemental Figure 2.** Project pipeline. First, adapters and barcodes were removed to obtain clean data. Second, Salmon and STAR+RSEM, two methods for quantification, were used. Third, after quantification, gene Venn diagram, PCA analysis, correlation stat, cluster analysis, and differential expressed analysis (by using DEseq2 and EdgeR together) were performed. Finally, pathway analysis was performed. **Supplemental Table 1.** MAPK pathway-related genes significantly differentially expressed between *Sirt2*-expressing cells and *Sirt2*-deficient cells. **Supplemental Table 2.** Calcium pathway-related genes significantly differentially expressed between *Sirt2*-expressing cells and *Sirt2*-deficient cells.

**Additional File 2 **Potential targets of deferential expressed genes with fold change > 10 and FDR < 0.1 in comparisons of *Sirt2*-expressing cells vs *Sirt2-*deficient cells; *Sirt2*-expression (cisplatin treated vs untreated) cells and *Sirt2*-deficient (cisplatin treated vs untreated) cells.

## Data Availability

The datasets supporting the conclusions of this article are included within the article (and its additional files).

## Abbreviations

ABCC9: ATP-binding cassette subfamily C member 9
AGTR2: Angiotensin II receptor type 2
BMP7: Bone morphogenetic protein 7
CAMK2A: Calcium/calmodulin dependent protein kinase II alpha
CAMK2D: Calcium/calmodulin dependent protein kinase II delta
CAMK2G: Calcium/calmodulin dependent protein kinase II gamma
Ctrl: Control
FC: Fold change
FDR: False discovery rate
KCNJ8: Potassium voltage-gated channel subfamily J member 8
KO: Knockout
PCA: Principal component analysis
QC: Quality control
Res: Rescue
Sirt2: Sirtuin 2
TPM: Transcripts per million
