# Supplementary material for: Sirt2-associated transcriptome modifications in cisplatin-induced neuronal injury
Source: BMC Genomics. 2020 Mar 2;21:192. doi: 10.1186/s12864-020-6584-2 (PMC7053098; doi:10.1186/s12864-020-6584-2)
Supplement: Supplementary file 1 — Additional file 1: Supplemental Figure 1. Timeline of cell differentiation. Samples were divided into 6 groups (Sirt2/Res, Sirt2/Ctrl, Sirt2/KO, Sirt2/Res + Cis, Sirt2/Ctrl+Cis, or Sirt2/KO + Cis). Cells were differentiated with Forskolin for 12 h and treated with cisplatin for another 24 h. After that, RNA was harvested. Supplemental Figure 2. Project pipeline. First, adapters and barcodes were removed to obtain clean data. Second, Salmon and STAR+RSEM, two methods for quantification, were used. Third, after quantification, gene Venn diagram, PCA analysis, correlation stat, cluster analysis, and differential expressed analysis (by using DEseq2 and EdgeR together) were performed. Finally, pathway analysis was performed. Supplemental Table 1. MAPK pathway-related genes significantly differentially expressed between Sirt2-expressing cells and Sirt2-deficient cells. Supplemental Table 2. Calcium pathway-related genes significantly differentially expressed between Sirt2-expressing cells and Sirt2-deficient cells. [file 12864_2020_6584_MOESM1_ESM.docx]

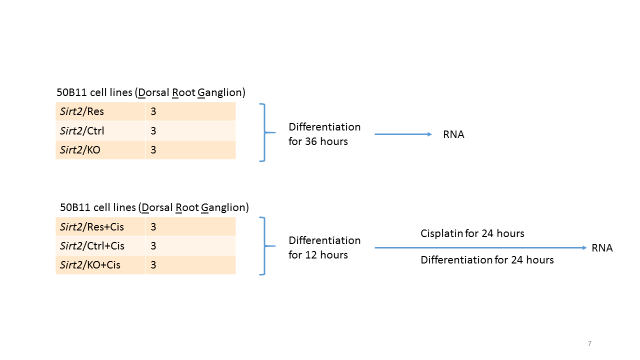


**Supplemental Figure 1.** Timeline of cell differentiation. Samples were divided into 6 groups (Sirt2/Res, Sirt2/Ctrl, Sirt2/KO, Sirt2/Res+Cis, Sirt2/Ctrl+Cis, or Sirt2/KO+Cis). Cells were differentiated with Forskolin for 12 hours and treated with cisplatin for another 24 hours. After that, RNA was harvested.


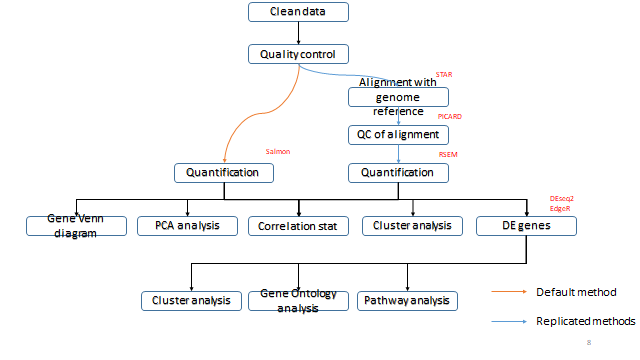


**Supplemental Figure 2.** Project pipeline. First, adapters and barcodes were removed to obtain clean data. Second, Salmon and STAR+RSEM, two methods for quantification, were used. Third, after quantification, gene Venn diagram, PCA analysis, correlation stat, cluster analysis, and differential expressed analysis (by using DEseq2 and EdgeR together) were performed. Finally, pathway analysis was performed.**Supplemental Table 1**. MAPK pathway-related genes significantly differentially expressed between Sirt2-expressing cells and Sirt2-deficient cells.

| Symbol | Fold Change | P-value |
| --- | --- | --- |
| *Bmp6* | 2.01 | 1.52E-02 |
| *Bmp7* | 5.22 | 3.96E-11 |
| *Cacna1c* | 2.2 | 6.11E-03 |
| *Cacna1g* | 2.82 | 2.20E-12 |
| *Ccl5* | 4.89 | 1.26E-02 |
| *Creb5* | 2.43 | 2.35E-03 |
| *Cx3cl1* | 6 | 3.18E-04 |
| *Cxcl10* | 2.76 | 4.80E-02 |
| *Cxcl13* | 2.49 | 7.89E-05 |
| *Cxcl3* | 2 | 3.35E-03 |
| *Edn1* | 3.25 | 5.02E-04 |
| *Efna5* | 2.92 | 1.88E-05 |
| *Figf* | 31.57 | 2.08E-05 |
| *Hspa8* | 10.54 | 1.44E-34 |
| *Igf2* | 3.79 | 1.33E-41 |
| *Il1r1* | 2.73 | 8.18E-10 |
| *Il1rn* | 4.26 | 1.79E-11 |
| *Inhba* | 3.52 | 6.36E-14 |
| *Kit* | 3.76 | 8.53E-26 |
| *LOC100910771* | 138.43 | 6.37E-31 |
| *Map2k6* | 3.61 | 1.39E-02 |
| *Pdgfra* | 8.77 | 7.76E-07 |
| *Rasgrp3* | 4.51 | 9.36E-20 |
| *Tgfb2* | 2.58 | 9.66E-20 |
| *Vcam1* | 3.9 | 7.39E-03 |

**Supplemental Table 2**. Calcium pathway-related genes significantly differentially expressed between Sirt2-expressing cells and Sirt2-deficient cells.

| Symbol | Fold Change | P-value |
| --- | --- | --- |
| *Atp2b4* | 2.47 | 7.44E-14 |
| *Plcd4* | 1.85 | 7.58E-02 |
| *Avpr1a* | 15.75 | 5.88E-05 |
| *Orai2* | 2.22 | 8.79E-17 |
| *Plcd4* | 1.85 | 7.58E-02 |
| *Atp2a3* | 4.41 | 5.93E-05 |
| *Grpr* | 4.77 | 8.99E-02 |
| *Ryr1* | 27.13 | 2.67E-02 |
